# 1941-2023 overall annual intensity indicator data for grapevine pests and diseases over three French vineyard regions

**DOI:** 10.1016/j.dib.2025.112397

**Published:** 2025-12-16

**Authors:** Lionel Delbac, Nathalie Smits, Anne Mérot, Marc Fermaud

**Affiliations:** a INRAE, BSA, ISVV, UMR 1065 SAVE, Villenave d'Ornon, France; b INRAE, CIRAD, Institut Agro, UMR 1230 ABSys, Montpellier, France

**Keywords:** Grapevine, Agricultural warning services, Grading system, Agricultural history, Crop protection

## Abstract

Since the beginning of the 20th century, the official French agricultural warning service (Ministry of Agriculture) has published weekly reports and annual summaries of key pest and disease pressures. The summaries were based on a large number of plots, including untreated ones, located in many different regions, with different local editions in every region. They constitute a highly valuable body of literature on pest and disease presence and overall damage, notably in vineyards. We used this literature to develop a textual analysis and build an integrative grading system for assessing a posteriori annual pest occurrence and damage intensity over an extended period (1941 to 2023) in the Bordeaux, Champagne and Vaucluse wine-growing regions. To reconstruct the pest and disease occurrence and intensity over time in the three regions, we established a long-term database of annual grades. The various grapevine diseases include notably downy and powdery mildews, black rot, rotbrenner and gray mold and, for the phytophagous insects, European vine moth (*Lobesia botrana*) and vine moth (*Eupoecilia ambiguella*). This tool can be very useful for characterizing the epidemiological status of various years or vintages, and analyzing long-term trends *versus* more isolated events. This will allow us to better describe and understand historical pest and pathogen temporal dynamics and link them to biotic and/or abiotic contexts. This will be helpful for anticipating needed advances in grapevine protection against quantitative and/or qualitative loss and for adapting viticulture to global changes including climatic, regulatory and marketing dynamics*.*

## Graphical abstract

**Figure:**
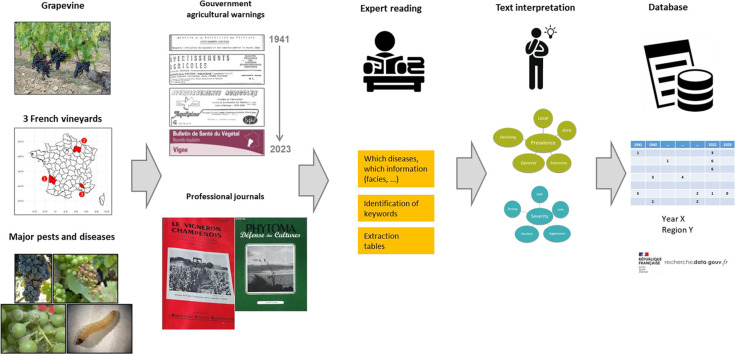

Specifications Table

**Table:** 

Subject	
Specific subject area	Agricultural Sciences: Agronomy and Crop Science: Plant Protection and Integrated Pest Management
Type of data	Table
Data collection	The data includes an integrated system, developed specifically for this purpose, to classify annual pest presence and damage intensity over a long period, and the corresponding bibliographical references. This was generated using textual analyses of periodic reports and annual summaries from the official French agricultural service analyzed to establish an annual grade for five major diseases and two key pests. The database cover eighty-three years of disease and pest presence and intensity in three French wine-growing regions.
Data source location	Institution: INRAE (Institut National de Recherche pour l'Agriculture, l'alimentation et l'Environnement), Ministère de l'Éducation nationale, de l'Enseignement supérieur et de la Recherche, Ministère de l'Agriculture et de la Souveraineté alimentaire City: Paris Country: France
Data accessibility	Repository name: Recherche Data Gouv repository https://recherche.data.gouv.fr/ Data identification number: https://doi.org/10.57745/HM3HBE Direct URL to data: https://entrepot.recherche.data.gouv.fr/dataset.xhtml?persistentId=doi:10.57745/HM3HBE
Related research article	none

## 1 Value of the Data

• This dataset constitutes the first use, at least in French viticulture, of a long-term series of multi-pathogen pressure indicators, based on the analysis of plant health reports.
• This approach will allow us to better explain past fluctuations in long-term epidemics and infestations.
• These data are useful in understanding the impact of cultural and/or regulatory changes.
• The potential evolution of pathogens and pests according to different scenarios of future climatic evolution should be modelized using this database.

## 2 Background

This database was built using a semi-quantitative and statistical description of implicit expert knowledge, a process known as elicitation [1]. The information we used regarded grapevine pests and diseases and was published in agricultural warning bulletins over several decades. To create the database, we used a complete and easily reusable scale, with iterative and empirical methods. The development of this new grading system allowed us to easily assess and compare the prevalence (local/general within the region considered) of epidemics for every key pathogen or pest considered. In addition, this scale makes it possible to distinguish between low-pressure and high-pressure years, notably by considering the extreme ends of the scale. The long-term records of these key pests and pathogens will also be investigated regarding how they relate to changes in viticultural practices and agroclimatic parameters.

## 3 Data Description

The dataset contains annual pressure indicators for 7 diseases or pests in 3 French wine-growing regions over a temporal analysis of 83 years from 1941 to 2023 (Table 1). However, not all the regions have the same number of records over time for a given disease or pest: the annual grades obtained are 489, 448 and 360 respectively for the Bordeaux, Champagne and Vaucluse regions.

**Table 1:** Descriptive data of annual grades obtained by disease or pest for each region over the period 1941 to 2023.

	Annual grades	Downy mildew	Powdery mildew	Black Rot	Gray mold	Rotbrenner	European vine moth	Vine moth
Bordeaux	Total computed	83	83	82	77	NA	83	81
	Minimum value	0	0	0	0	NA	0	0
	Maximum value	6	6	6	6	NA	6	3
	Median value	2	2	2	2	NA	2	0
Champagne	Total computed	64	64	64	64	64	64	64
	Minimum value	0	0	0	0	0	0	1
	Maximum value	6	6	2	6	3	3	6
	Median value	3	2	0	3.5	1	2	4
Vaucluse	Total computed	69	68	56	51	NA	68	48
	Minimum value	1	1	0	0	NA	1	1
	Maximum value	6	6	4	6	NA	6	6
	Median value	4	3	1	2	NA	2	2

NA: missing values.

**Fig. 1:**
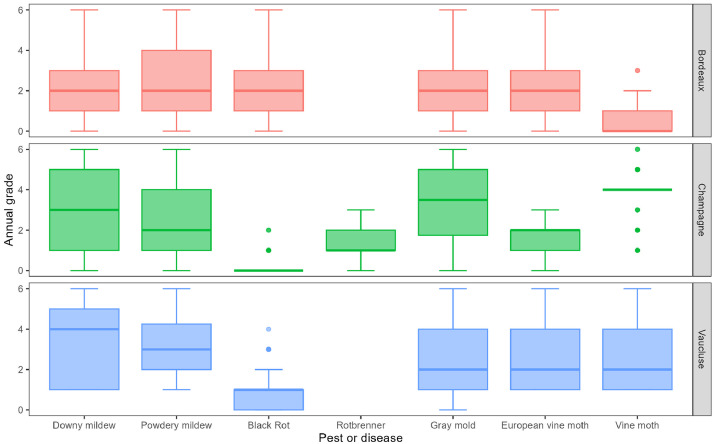
Summary graph displaying the dataset of annual grades for each of the 3 regions (Bordeaux, Champagne and Vaucluse) and for each disease and pest assessed: downy mildew, powdery mildew, black rot, rotbrenner, gray mold, European grapevine moth and vine moth.

Fig. 1 provides an overview of the database. In the Bordeaux region for downy mildew, the disease is always present, but tends to be localized, with more moderate forms than over regions. For the other diseases and pests, the reported data range from no presence at all to significant and widespread damage, except for vine moth, which is always less present.

In the Champagne region, downy mildew, gray mold and vine moths are the regularly occurring diseases and pest causing heavy damage. Black rot was absent for a long time, but was reported locally starting in the late 1990s with low damage. European vine moth, on the other hand, was localized and sometimes very severe.

In Vaucluse, mildews are the main recurring diseases, often reported as widespread and/or very severe. Rotbrenner was not recorded and black rot is reported not severe.

**Fig. 2:**
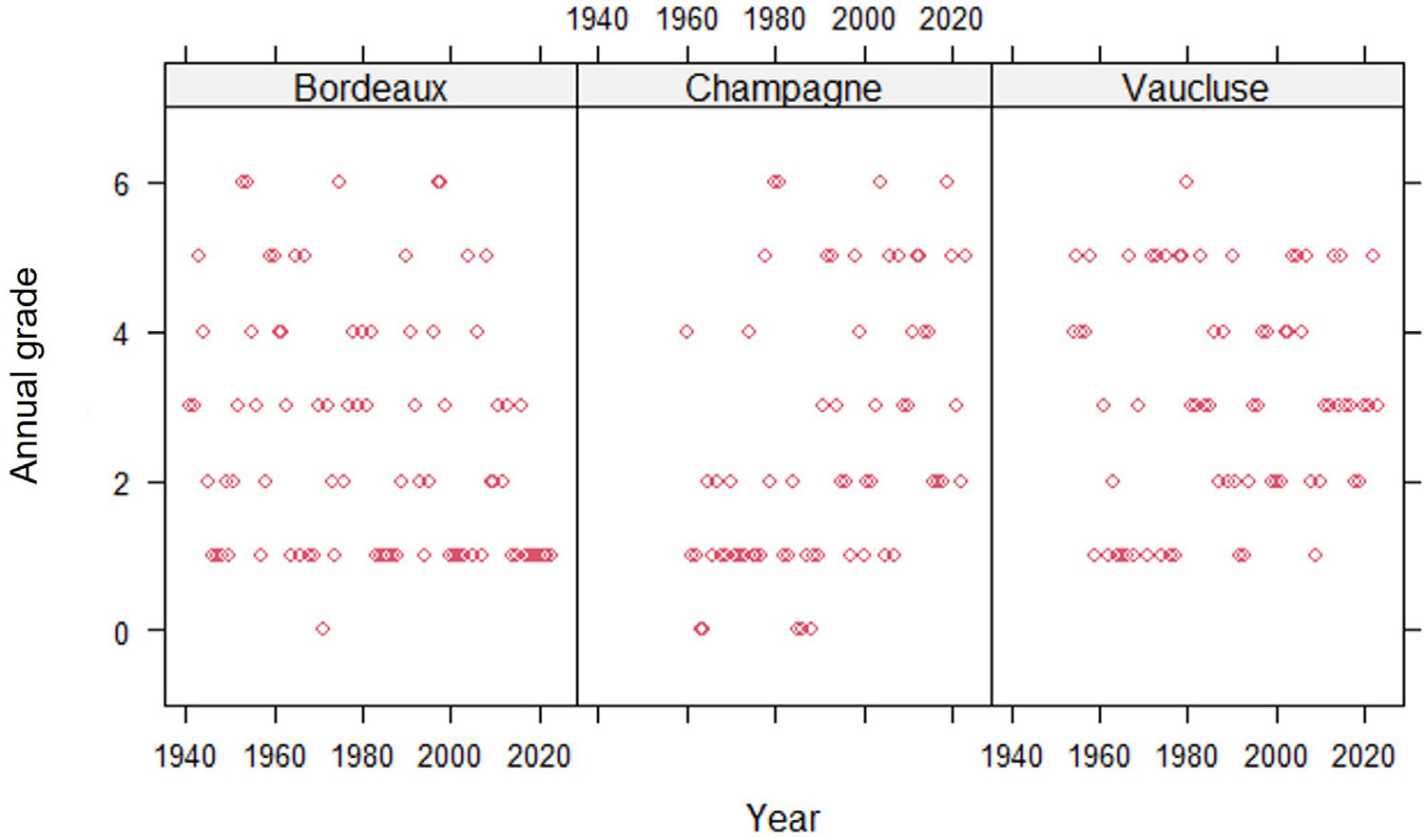
Historical trend in the annual grade assessed by the global index developed for each of the 3 regions (Bordeaux, Champagne and Vaucluse) for powdery mildew.

**Fig. 3:**
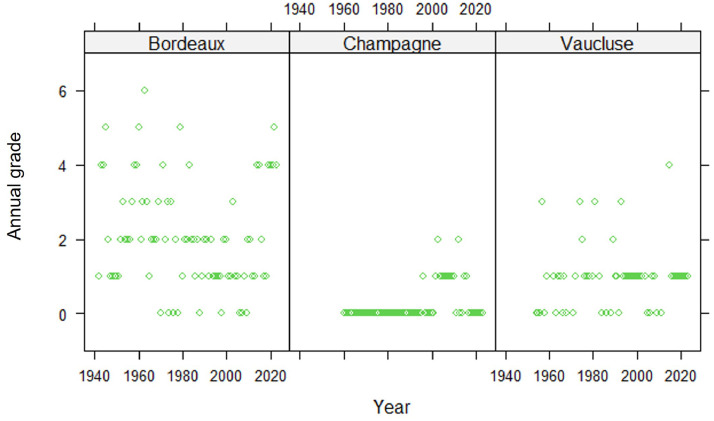
Historical trend in the annual grade assessed by the global index developed for each of the 3 regions (Bordeaux, Champagne and Vaucluse) for black rot.

**Fig. 4:**
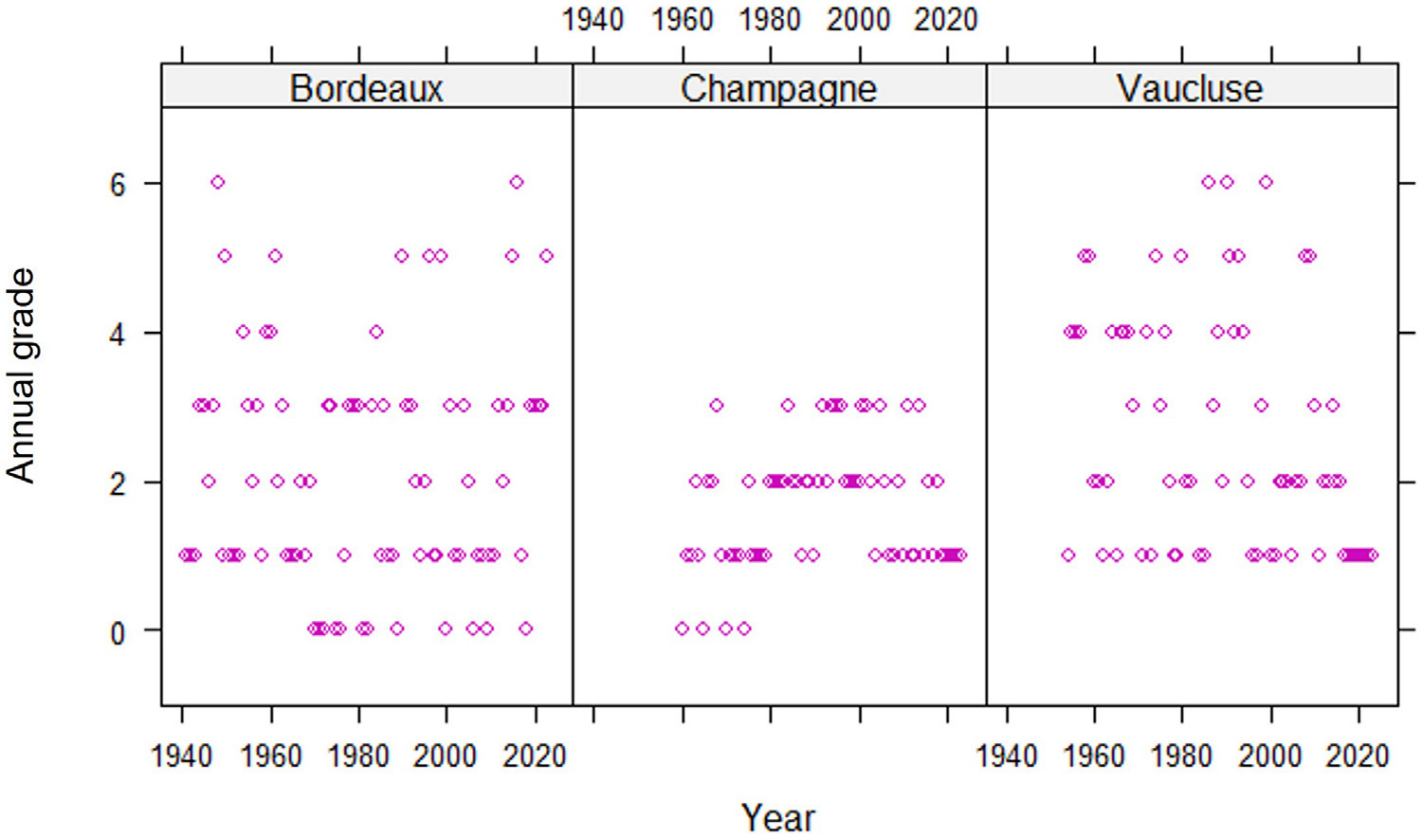
Historical trend in the annual grade assessed by the global index developed for each of the 3 regions (Bordeaux, Champagne and Vaucluse) for European grapevine moth.

The three following case studies (Fig. 2, Fig. 3, Fig. 4) show in more details the temporal dynamics in the three regions of powdery mildew, black rot and European grapevine moth, respectively.

For powdery mildew (Fig. 2), the temporal dynamics shows significant variations from year to year, ranging from no occurrence (rarely) to widespread distribution with very severe forms of the disease. There are marked significant differences in temporal dynamics, in both Champagne and Vaucluse regions, where epidemics are becoming increasingly widespread, *versus* the Bordeaux region where severity is declining and outbreaks are more localized.

Black rot is a disease with very different patterns according to the region (Fig. 3). In Bordeaux, epidemics are often observed, usually locally and with moderate severity. The rare widespread epidemics cause little damage. In Champagne, the disease has been observed more recently, again locally, without causing any damage. Finally, in Vaucluse, epidemics are recurrent but localized and of low intensity.

For European grapevine moth (Fig. 4), population dynamics vary between regions. While populations are smaller and more localized in Champagne, they are more widespread in Vaucluse. In both regions, an increasing localized pest pressure is noticeable. In the Bordeaux region, the annual situations vary greatly, but here too, infestations are becoming increasingly localized.

## 4 Experimental Design, Materials and Methods

### 4.1 Selection of the study regions

**Fig. 5:**
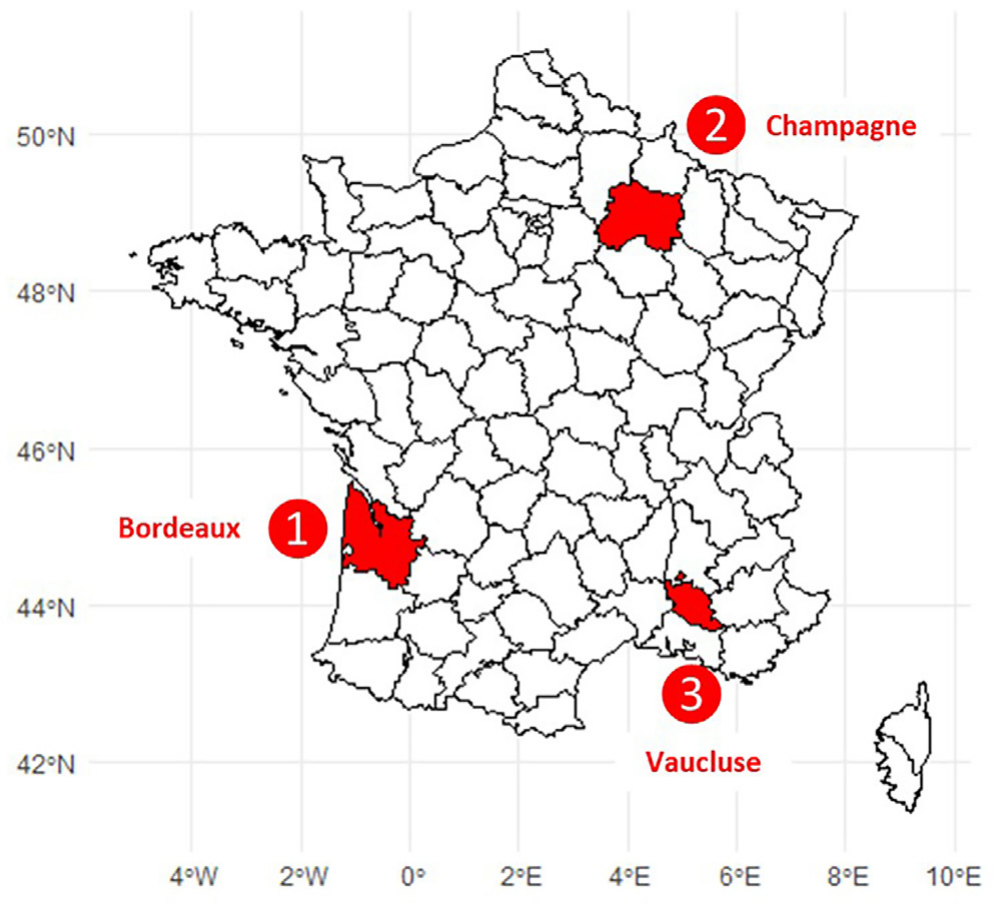
Location of the 3 French wine-growing regions studied. The emblematic departments in these vineyard regions are coloured red with: 1) Bordeaux (Gironde Department); 2) Champagne (Marne Department); and 3) Vaucluse (Vaucluse Department).

The historical phytosanitary analyses were carried out for three French wine-growing regions (Fig. 5) with very different practices, soil and climatic conditions and production methods:

- The Bordeaux viticultural region, known for its red *Appellation d’Origine Contrôlée* (AOC, refers to standards set for wines made in France) wines with aging capacity, is located in a temperate oceanic climate, *i.e.,* a humid temperate climate characterized by mild, wet winters and cooler summers than in the Mediterranean one. The subsoil is composed of sediments with clay-limestone soils of neutral or alkaline pH, or gravel soils that are more acidic. The relatively flat vineyards cover an area of almost 100,000 hectares, predominantly planted with black grape varieties, at an average density of 5000 to 7500 vines per hectare, trellised, with an average yield of 50 hl per hectare.

- The Champagne viticultural region, known for their sparkling high-quality AOC wines, are located in a continental climate under a partial oceanic influence, characterized by cool winters, mild summers and frequent but not very abundant rainfall dispersed throughout the year. The soil is characterized by chalk, marl and sand. The fairly steep vineyards cover an area of almost 30,000 hectares, dominated by black grape varieties, planted at a high density of 8000 to 10,000 vines per hectare, trellised, with an average yield close to 100 hl per hectare.

- The Vaucluse viticultural region is part of the *Côtes-du-Rhône* AOC. It is located in a typical warm-temperate Mediterranean climate, characterized by hot, dry summers and mild, wet winters. One particular feature is the dominance of the very drying Mistral north wind. The soils are characterized by rolled pebbles on clay or sandy soils. The vineyards stretch over the plains and gently sloping hills bordering the river Rhône, covering an area of around 70,000 hectares and producing around 50 hl per hectare. The vineyards are mainly planted with black grape varieties, with a wide combination of varieties, some of which are still trained in gobelets, while the majority are trellised. Planting density is around 4000 vines per hectare.

### 4.2 Selection of pests and diseases studied

The present study focuses on the vineyard’s main phytosanitary problems, which are encountered at a national level to different extents [2,3,4].

In both regions, a total of 7 major species of pathogen and moth were studied:

- downy mildew = *Plasmopara viticola* (Berk. & M.A. Curtis) Berl. & De Toni, 1888 was reported for the first time in 1878 in France near Bordeaux. In the following years, massive epidemics were deemed responsible for the loss of about 50 % of the French grapevine production. At the beginning of the 20th century, reports of yield loss due to this disease varied widely, cycling between years of massive loss and years virtually free of the disease.

- powdery mildew = *Uncinula necator* Schwein. 1834 was the first American pathogen to be introduced in Europe. The disease was reported in France in 1848. Within a few years, epidemics were reported all across the country, causing heavy losses. The discovery that sulfur could be used against the disease, and the development of methods for its large-scale application, brought the disease under control.

- black rot = *Guignardia bidwellii* (Ellis) Viala & Ravaz 1892 was first identified in 1885 in Southeast France. It spread over Southwest France, causing devastating epidemics, at the end of 19th century. Indigenous to North America, the disease can constitute a serious threat in wet oceanic regions with persistently damp conditions, moderate temperatures, and warm weather. In modern times, the disease is mostly controlled by several groups of fungicides.

- rotbrenner = *Pseudopezicula tracheiphila* (Müll.-Thurg.) Korf & W.Y. Zhuang is a fungus of European origin. It causes a disease that is relatively rare in French vineyards, but is most prevalent in Northeast France. This fungus invades the vascular system of the grapevine, causing symptoms such as leaf and fruit drop, leading to destroyed berries in severe cases.

- gray mold = *Botrytis cinerea* Pers. 1794 is a fungus that has been documented for a long time affecting numerous plant species, and is found in vineyards throughout the world. The regions where the disease is most severe are characterized by moderate temperatures, and rainfall or extended periods of high humidity between veraison and harvest. Yield reductions are often associated with pre- or post-harvest berry rot, and are sometimes due to damaged flower clusters early in the season. Quality loss results from the modified chemical composition of diseased berries.

- European grapevine moth = *Lobesia botrana* (Denis & Schiffermüller 1775) appeared on grapevines at the beginning of the 20th century. At the time of harvest of infected berries, it was common to find a lot of caterpillars on the racks or presses. In wet years, the subsequent *Botrytis* attacks were especially problematic, especially in grape varieties with high cluster compactness, and made the winemaking process more complicated. The absence, for a very long time, of sufficiently effective insecticides explains the extraordinary outbreaks of the moth. The discovery of new insecticides and improvements in treatment equipment have significantly reduced the negative economic impact of the insect since the end of World War II.

- vine moth = *Eupoecilia ambiguella* (Hübner 1796) was one of the earliest identified vineyard pests in France, causing historically considerable losses in the vineyards of Northeast France. The insect is a widespread pest all over France, although it is considered to be more of a problem in northern regions, and causes varying economic impacts according to the region.

### 4.3 Materials

For this study, we used the collection of phytosanitary warning bulletins created by French regional authorities (Ministry of Agriculture) [5,6]. The data collected in these historical sources is consistent over a long period of time, and at the wine-growing scale, making them ideal for our purposes. They were designed to provide regulatory information, to help monitor plant protection and to help growers correctly apply pesticide treatments [7]. The bulletin content was based on observations, trapping and/or modelling [5,7]. From the outset, the regional reports were sent to growers several times within each season and complemented with annual syntheses. The authors of these reports also included strategic and practical advice. Before the Second World War, the first bulletins sent out were very concise and were comprised of a very limited number of sentences, mostly advising on the need for pesticide treatments. Gradually, the reports expanded to a more comprehensive and analytical several-page format. For each pest, prevalence, often according to some defined sub-regions, and severity level were recorded. To complement this data, we also used monthly or annual summaries published in technical journals distributed to technicians and winegrowers, either regionally (*Le Vigneron Champenois*) or nationally (*Phytoma-La Défense des Végétaux*). These summaries are written by experts involved in agricultural warning services and enabled us to compensate for information lacking in library databases.

Across the 3 regions, we tried to collect documents covering the period from 1941 to 2023. However, it was not possible to find information spanning the entire period for all three regions (Table 2), so the most comprehensive data spans from 1960 to 2023. The documents were better preserved in Bordeaux and we did not need to supplement them. We used then mostly the annual summaries written at the end of each year. In Champagne, the archive collection was more limited, but we were able to access the collection of the regional journal *Le Vigneron Champenois* in which Ministry technicians wrote annual or seasonal summaries together with specialized journalists. In Vaucluse, the documentation is intermediate, but there is a gap in the archives for 1970. We were unable to fill this gap due to the absence of a regional technical journal and the absence of national documents. In the end, 305 documents were retrieved and studied.

**Table 2:** Type and number of documents analyzed to build the database and period in each of the 3 regions studied.

Region	Period	Periodic reports	Annual reviews	Regional technical journal	National technical journal	Total
Bordeaux	1941 to 2023	19	65	0	3	87
Champagne	1960 to 2023	12	40	96	0	148
Vaucluse	1954 to 1969 & 1971 to 2023	25	43	0	2	70
Total		56	148	96	5	305

### 4.4 Methods

The process used can be summed up in the following three steps: i) collection of bibliographical data; ii) extraction of the data from the plant health bulletins; and iii) creation of the database. The main goal was to convert the qualitative written information into standardized numerical grades. Careful reading of the bulletins revealed the need to translate the information into a semi-quantitative scale, allowing analyses and comparisons and sometimes necessitating arbitrary classifications. Zwankhuizen and Zadoks [8] used a five-grade scale to address the overall annual intensity of potato disease. We used a similar scale in grapevine for a multi-pest damage indicator [9]. However, this scale did not allow us to characterize most of the epidemical cases recorded in the warning literature. Thus, a new seven-grade scale was designed to distinguish between low-and high-pressure years, including exceptional and extreme years for each pest considered [10] (Table 3). These semi-quantitative grades are a combination of occurrence/prevalence data and associated overall damage/severity. They were elaborated for every pest and disease, by extracting selected keywords from the texts. We collected all the words or key parts of sentences, describing the observed prevalence on one hand, and the severity on the other hand, of the pest. We then translated these textual elements into a severity and a prevalence level, and constructed an aggregated scale of these two levels to obtain the final overall annual grade. Repeating these steps every year allowed us to build time series of pest or disease level in the studied important French winegrowing regions.

**Table 3:** Semi-quantitative grading scale of annual overall pest intensity: 0 no epidemic; grades 1 to 3, local epidemics; grades 4 to 6, general epidemics.

	OVERALL SEVERITY				
		Null	Low	Medium	High
PREVALENCE/DISTRIBUTION	Null	**0**: The pest/disease not present in the vineyard	-	-	-
	Local	-	**1**: Localized, almost non-existent, very weak and/or limited damage	**2**: Localized, medium severity/damage (ex. in susceptible sites/untreated areas only)	**3**: Localized, severe, high intensity/damage (ex. in susceptible sites/untreated areas only)
	General	-	**4**: Widespread, low intensity/damage (only limited areas show no signs of the pest/disease)	**5**: Generalized or high pest/disease pressure or high pressure/widespread damage	**6**: Widespread, very high/exceptional pressure, significant damage

We have built up a database for each region studied and for each disease or pest considered (Table 4). If the information is not clearly interpretable (for example, the report does not specify which of the 2 moth species was recorded) the grade is not awarded and NA (not assessed) is given instead. If a disease or pest was not mentioned, NA is also awarded. In total for the 7 diseases and pests and the 3 regions considered from 1941 to 2023, 1297 annual grades were awarded, compared with 447 NA.

**Table 4:** Description of the long-term database of annual classes of grapevine pest and disease data files.

File Name (Size)	File Format (size)	Description
Read_me.txt	ASCII (3.6 KB)	Metadata file describing the data contained in the files
Long-term evolution of a global annual intensity indicator for grapevine pests in three French vineyards.tab	ASCII file (7.8 KB)	1941–2023 annual grade obtained by disease or pest for each region
Metadata Long-term evolution of a global annual intensity indicator for grapevine pests in three French vineyards.tab	ASCII file (0.6 KB)	Metadata Long-term evolution of a global annual intensity indicator for grapevine pests in three French vineyards
List of bibliographical references used.csv	ASCII file (56.8 KB)	List of bibliographical references used
Metadata list of bibliographical references used.csv	ASCII file (0.6 KB)	Metadata list of bibliographical references used
Semi-quantitative scale of annual overall pest intensity.csv	ASCII file (0.7 KB)	Formula of the semi-quantitative scale used of annual overall pest intensity

## Limitations

Not applicable.

## Ethics Etatement

This research meets the ethical requirements for publication in Data in Brief. This work dos not involve studies with animals and humans, or data collected from social media platforms.

## CRediT authorship contribution statement

**Lionel Delbac:** Methodology, Resources, Validation, Formal analysis, Data curation, Writing – original draft, Visualization. **Nathalie Smits:** Conceptualization, Supervision, Funding acquisition, Project administration, Methodology, Resources, Validation, Formal analysis, Data curation, Visualization, Writing – review & editing. **Anne Mérot:** Conceptualization, Supervision, Funding acquisition, Project administration, Methodology, Resources. **Marc Fermaud:** Conceptualization, Supervision, Funding acquisition, Project administration, Methodology, Resources, Validation, Formal analysis, Writing – review & editing.

## Data Availability

Recherche Data Gouv repositoryLong-term temporal dynamics of an overall annual intensity indicator of grapevine pests and diseases in three French vineyards (Original data). https://entrepot.recherche.data.gouv.fr/dataset.xhtml?persistentId=doi:10.57745/HM3HBE
